# OpenTein: a database of digital whole-slide images of stem cell-derived teratomas

**DOI:** 10.1093/nar/gkv1096

**Published:** 2015-10-22

**Authors:** Sung-Joon Park, Yusuke Komiyama, Hirofumi Suemori, Akihiro Umezawa, Kenta Nakai

**Affiliations:** 1 Human Genome Center, The Institute of Medical Science, The University of Tokyo, 4-6-1 Shirokanedai, Minato-ku, Tokyo 108-8639, Japan; 2 Department of Embryonic Stem Cell Research, Institute for Frontier Medical Sciences, Kyoto University, 53 Kawahara-cho, Shogoin, Sakyo-ku, Kyoto 606-8507, Japan; 3 Department of Reproductive Biology, National Institute for Child Health and Development, 2-10-1 Okura, Setagaya-ku, Tokyo 157-8535, Japan

## Abstract

Human stem cells are promising sources for regenerative therapy. To ensure safety of future therapeutic applications, the differentiation potency of stem cells has to be tested and be widely opened to the public. The potency is generally assessed by teratoma formation comprising differentiated cells from all three germ layers, and the teratomas can be inspected through high-quality digital images. The teratoma assay, however, lacks consistency in transplantation protocols and even in interpretation, which needs community-based efforts for improving the assay quality. Here, we have developed a novel database OpenTein (Open Teratoma Investigation, http://opentein.hgc.jp/) to archive and freely distribute high-resolution whole-slide images and relevant records. OpenTein has been designed as a searchable, zoomable and annotatable web-based repository system. We have deposited 468 images of teratomas derived by our transplantation of human stem cells, and users can freely access and process such digital teratoma images. Approximately, the current version of OpenTein responds within 11.2 min for processing 2.03 gigapixel teratoma images. Our system offers valuable tools and resources in the new era of stem cell biology.

## INTRODUCTION

Human embryonic stem cells (hESCs) and human induced-pluripotent stem cells (hiPSCs) are promising sources for stem cell biology and regenerative therapy. Prior to the practical use of the stem cells, it is important to characterize their pluripotency *in vivo* for safety and success in further applications (1,2). In general, the transplantation of human stem cells into immuno-deficient animals is used to demonstrate the capacity of cell pluripotency, which forms teratomas comprising differentiated cells from all three germ layers (3–5). Recently, high-quality microscopes providing digital whole-slide images (WSIs) facilitate the inspection of teratoma histologic features (6), and WSIs are becoming a highly valuable resource because they also offer insights into the mechanisms of tissue development (7).

Unfortunately, while the teratoma assay is considered as the ‘*gold standard*’ for pluripotency tests, this assay lacks consistency in experimental protocols, leading to contradictory assessments and poor reproducibility (1,8). This is due in part to the variability of experimental parameters (e.g. the number of cells injected, injection sites and growth conditions) that potentially affect the results of histopathological analysis. In addition, journal publications that detail only a representative image of teratomas cannot bring out the potential of teratoma formation assay; it is unclear whether derivatives of all germ layers are found in the same section of teratomas. Although efforts have been made to standardize the teratoma assay (2,8,9), no community-based attempts have been made to archive and freely distribute the protocols, resultant WSIs and expert histological assessments. As learnt from public repositories, such attempts enhance not only the fundamentals of qualitative and quantitative assays but also transparent reproducibility and accessibility (10–12).

Here, we have developed a database OpenTein (Open Teratoma Investigation) having web-based features as a public repository system. Like other repositories (13), OpenTein requires user registration for manipulating the teratoma assays including WSIs and relevant records. Along with assigning unique identifiers, OpenTein automatically creates web pages containing zoomable and annotatable high-resolution digital images in a vendor-independent fashion. Thereby, users can easily share and promote their teratoma assays through the internet. We have currently deposited 468 assays derived by our transplantation (4,5), consisting of 52 for hESCs and 416 for hiPSCs. Anyone can freely access the valuable resource at http://opentein.hgc.jp.

## SYSTEM OVERVIEW

### Interactive web-based platform

OpenTein has been designed to make WSIs accessible to users who lack computational resources; the file size of WSIs is massive ranging from several hundred megabytes to several gigabytes (14), which impacts the storage capacity of cost-intensive commercial server systems. In parallel, OpenTein has been intended to offer users traceable and annotatable teratoma assays. As shown in several studies (14,15), the WSIs can be systematically managed by using open-source libraries. Thus, such well-organized resources can be used for various purposes; e.g. quantitative assays and the automatic identification of histological features (16,17).

**Figure 1.**
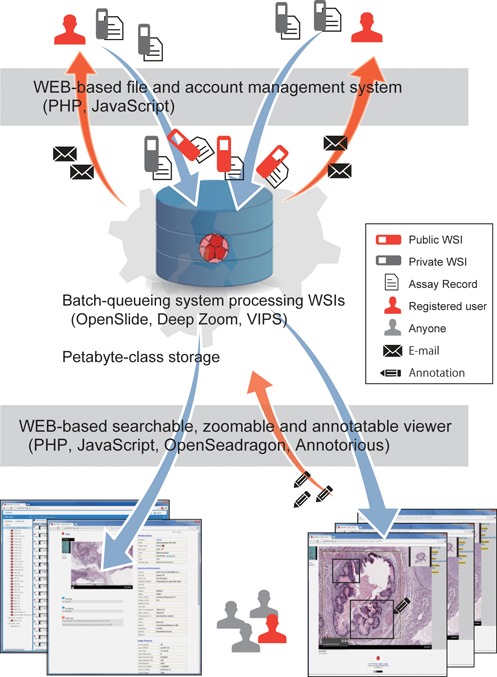
A schematic representation of OpenTein as a repository system of the teratoma assay.

As illustrated in Figure 1, OpenTein that has been installed on a supercomputer with petabyte-class storage provides the interactive web-based interface for freely registering accounts and easily depositing the assays. Registered users can specify the sharing mode of uploaded assays; i.e. public or private (hidden from other users). For individual public assays, OpenTein assigns a unique accession number beginning with ‘OTi’, which provides accessibility if the accession numbers are cited in journal publications, for example. In addition, to help users share comments and annotations, OpenTein embedded the Facebook comment plugin and equipped an online annotation tool. When the processing of uploaded WSIs was finished and annotations were modified by other users, OpenTein sends e-mail messages to owners.

### Key features

OpenTein was built by the programming language PHP and JavaScript. The system automatically processes WSIs by submitting batch jobs to create multilayered pyramid images with smaller tile images, which is the most time-consuming step and allows users to pan and zoom at various magnifications by manipulating the mouse of a computer. This processing utilizes vendor-neutral image software, i.e. OpenSlide (18) running by VIPS (ver. 7.42, http://libvips.blogspot.jp/). We currently accept the following file formats; Aperio SVS, Hamamatsu NDPI, Leica SCN and generic TIFF. The web-based WSI viewer has been implemented with open-source JavaScript OpenSeadragon (https://openseadragon.github.io/) and Annotorious (http://annotorious.github.io/) coupled with in-house developed APIs.

**Table 1.** List of features available for each user type

	Registered user		Non-registered user
	Public	Private	Public
Search	Yes	Yes	Yes
Browse	Yes	Yes	Yes
Annotation	Yes	Yes	Yes*
File download	Yes	Yes	Yes
File upload	Yes	Yes	
E-mail notice	Yes	Yes	
Delivery	Yes	Yes	
Accession Number	Yes		

*not saved in the OpenTein database.

As summarized in Table 1, anyone can explore and access public assays through search and annotation options, although annotations by non-registered users are not stored in OpenTein to prevent vandals. Therefore, the user registration with a valid e-mail address is important to effectively use our system. In addition, we prepared a delivery function that a registered user can send WSIs to another registered user. This feature helps seamless collaboration of users. For instance, researchers who have imaging facilities digitalize teratomas and pass WSIs to sample providers who do not have such facilities.

It is important to note that for ethics’ sake OpenTein monitors any events occurring on all deposited assays even on private assays.

## WEB INTERFACE

**Figure 2.**
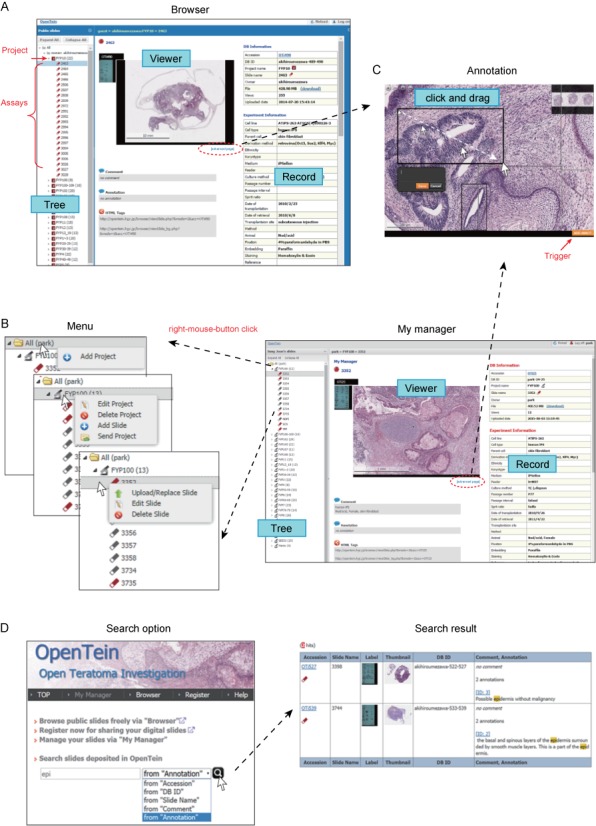
Screenshots of the web-based interface. (**A**) Anyone can explore the public assays through ‘Browser’ implemented with a hierarchical data tree. (**B**) Registered users can work through the interface ‘My manager’. (**C**) The annotation can be simply done by drawing a rectangle for a region of interest. (**D**) The search option enables easy access to deposited assays.

Since a teratoma assay often involves a series of WSIs, we manage users’ depositions as a hierarchical data tree that has clickable multilevel nodes; e.g. in the public interface ‘Browser’ (Figure 2A), ‘All’ (root node), ‘Owner’ (registered user ID), ‘Project’ (a group of assays) and assay (end node). For each of the registered users, we prepared a special workspace, namely ‘My manager’. In this workspace, users can control assays by clicking the right-mouse-button on the tree nodes after login to OpenTein (Figure 2B). Clicking the menu items ‘Add Slide’ and ‘Edit Slide’ (Figure 2B) shows pop-up windows that enable users to enter and modify the corresponding experimental details. The required experimental details have been chosen based on our empirical knowledge, which needs further community-based discussions for standardizing the minimum information (11,12) that must be reported for the interpretability and reproducibility.

For annotating WSIs, whenever the trigger button ‘Add annot.’ is available on the viewer, one can mark a region of interest by drawing a rectangle and then can comment it (Figure 2C). If an annotation was created by a registered user, OpenTein assigns a unique ID for it. Although the annotations are hyperlinked from Browser and My manager, they can be specified by the internet URLs. Alternative is the search option that allows users to query such as annotation from registered users, accession number, database ID, slide name described by owners and comment provided by owners (Figure 2D). Registered users can find private assays also by the search option after login.

Users can find more instructions from the FAQ (Frequently Asked Questions) website at http://opentein.hgc.jp/FAQ/faq.php.

## PROCESSING OF WHOLE-SLIDE IMAGE

### Impact of a tile size on storage

To help users quickly zoom in on different parts of the high-resolution image, OpenTein runs VIPS to process WSIs into small JPEG tiles in Deep Zoom format, and the tile size (e.g. 256 × 256 pixels) is an important factor for the performance of our system. By using the 468 WSIs in SVS file format we deposited to OpenTein, we confirmed that the total number of tiles created from single SVS file is depending on the original resolution and the tile size (Supplementary Figure S1A). The total file size of tiles became approximately 1.5 times larger than that of the original SVS file (Supplementary Figure S1B); on average, 590.7 megabyte (MB) SVS file is converted into 982.4, 900.4 and 873.6 MB tiles in 256, 512 and 1024 tile size, respectively. These results suggest that tiling WSIs into 256 × 256 pixel images requires huge file quotas, and that the requirement of storage capacity is not dramatically changed among different tile sizes. A bigger tile size (e.g. 1024 × 1024 pixels) will lead to dissatisfaction, since users need a longer time to load such heavy tiles. We set it to 512 in here.

### Response times

The processing of uploaded WSIs is executed on a system that has been built with 2 terabyte (TB) memory and 48 cores of 3.0 GHz Intel CPU E7-8857. As shown in Supplementary Figure S1C, WSI resolutions affect the response time that is the elapsed time from start to end of a submitted batch job. Although such response times can be reduced by dividing a process into several multiple tasks simultaneously running (i.e. threads), larger number of threads requires more memory that is almost constant against the WSI resolutions (Supplementary Figure S1D). We set the number of threads to 12 in here.

On average, the current version of OpenTein running with 12 threads that require 8.02 gigabyte (GB) memory responds within 11.2 min when one finished uploading a 2.03 gigapixel WSI (590.7 MB), and generates 9936.1 tiles in 512 × 512 pixels (900.4 MB in total file size).

## CONCLUSIONS

Although alternatives exist (e.g. gene expression profiling and the tetraploid complementation assay), the teratoma assay is widely used for proving pluripotency of human stem cells. To archive the teratoma assays and to share them enhance the fundamentals of effective pluripotency tests. For accomplishing this, we developed a database OpenTein that makes high-resolution WSIs and relevant notes uploadable, accessible and traceable to public users. In this study, we have deposited 468 WSIs delivered by our experiments, and anyone can use this resource through the user-friendly web interface.

For its further progress, community-based efforts are indispensable as expert histological assessments need the time and interpretative efforts. In this regard, to implement computational algorithms that aid the expert investigation to succeed is challenging but worthwhile. To improve its usability, we aim to further design web-based interfaces for several features, such as uploading multiple files at a time and annotating by various shapes and colors. Moreover, transferring data in human- and machine-readable formats is important to systematically manage the assays, which helps to guide the minimum information that ensures the interpretability and reproducibility. We believe that such well-structured records along with data sharing will enhance stem cell biology and regenerative therapy.

## SUPPLEMENTARY DATA

Supplementary Data are available at NAR Online.
